# Iron Levels in Bronchoalveolar Lavage Fluid of Hematological Patients with Suspected Invasive Pulmonary Aspergillosis and their Association with 12-week Mortality: A Retrospective Cohort Study

**DOI:** 10.1007/s11046-025-00934-w

**Published:** 2025-02-03

**Authors:** H. Lamberink, J. Heijmans, A. Wagemakers, K. van Dijk

**Affiliations:** 1 Department of Medical Microbiology and Infection Prevention, Amsterdam University Medical Centers, Amsterdam, the Netherlands; 2 Department of Hematology, Amsterdam University Medical Centers, Amsterdam, the Netherlands; 3 Department of Medical Microbiology and Infection Prevention, Onze Lieve Vrouwe Gasthuis, Amsterdam, the Netherlands

**Keywords:** Invasive pulmonary aspergillosis, Bronchoalveolar lavage, Iron concentration

## Abstract

**Objectives:**

Accuracy of diagnostic tests for invasive pulmonary aspergillosis (IPA) using bronchoalveolar lavage fluid (BALF) remains suboptimal. Elevated tissue iron in lung transplant and murine models is linked to invasive *Aspergillus* growth. This study examines the correlation between BALF iron levels, IPA, and 12-week mortality.

**Methods:**

We conducted a retrospective cohort study at a tertiary care center, including 100 BALF samples from patients with hematological malignancies and suspected IPA between 2014 and 2019. Data regarding iron concentrations, mycological tests, and 12-week mortality were analyzed.

**Results:**

Higher iron levels correlated with a greater likelihood of IPA based on EORTC/MSGERC 2020 definitions (*p* = 0.038). The ROC area was 0.648 (95% CI 0.531-0.764), with an optimal cut-off of 0.75 µmol/L to distinguish cases (27 probable and 0 proven IPA) from controls (56 possible and 17 no IPA), with sensitivity 76.9% and specificity 47.3%. Iron levels were positively correlated with higher fungal loads (galactomannan: Spearman’s ρ 0.323, *p* = 0.001; *Aspergillus* PCR Ct-values: ρ − 0.602, *p* = 0.002). A trend toward higher 12-week mortality was observed in patients with iron concentrations ≥ 0.90 µmol/L compared to lower levels (*p* = 0.086).

**Conclusions:**

BALF iron concentrations were highest in those with probable IPA, followed by possible IPA and lowest in patients without IPA, with higher iron levels also correlating with fungal loads and potentially with 12-week mortality. However, given the various potential confounding factors, further prospective studies are essential to establish causality. These findings warrant additional investigation into BALF iron as a potential marker for 12-week survival, but validation is necessary before considering it as a supplementary marker in the current EORTC/MSGERC 2020 classification for probable or possible IPA.

**Supplementary Information:**

The online version contains supplementary material available at 10.1007/s11046-025-00934-w.

## Introduction

Invasive pulmonary aspergillosis (IPA) has a high mortality rate of 30–70% [1, 2]. Severely immunocompromised patients, such as those who have undergone hematopoietic stem cell transplantation for hematological malignancies, face an IPA incidence of 6–16% [3, 4]. Recently, the European Organization for Research and Treatment of Cancer and the Mycoses Study Group Education and Research Consortium (EORTC/MSGERC) updated their consensus definitions for diagnosis of IPA [5]. These definitions comprise host factors, clinical criteria, and mycological criteria: direct microscopy, fungal culture, galactomannan index (GM) and *Aspergillus* polymerase chain reaction (PCR) on serum and/or bronchoalveolar lavage fluid (BALF). However, diagnostic sensitivities vary, with fungal culture showing < 11% [6, 7], GM index 70–90% [8, 9] and *Aspergillus* PCR 62–88% [10]. Given the serious impact of IPA in patients with hematological malignancies, there is a critical need for improved diagnostic tests.

Virulence and invasive growth of infectious microorganisms are influenced by various factors, of which available iron has previously been suggested to be a notable one [11, 12]. Higher serum iron and ferritin levels are associated with increased mortality in mucormycosis [13], and elevated tissue iron levels correlate with a higher prevalence of invasive fungal disease and acute rejection episodes in lung transplant patients [14–17]. Moreover, enhanced virulence of pulmonary *Aspergillus fumigatus* was linked to higher tissue iron concentrations from microhemorrhages in murine lung grafts [18].

This study investigated the correlation between BALF iron concentration and *Aspergillus* loads in patients suspected of IPA in the hematology department, assessing its potential as a diagnostic marker for IPA alongside the current EORTC/MSGERC 2020 criteria, and its predictive value for 12-week mortality.

## Methods

This retrospective, observational study was conducted in accordance with the STROBE criteria [19]. A waiver was obtained from the local Medical Ethics Review Committee (2019–090).

### Patient Population

BALF samples were collected from adult patients with hematological malignancies who underwent BAL for suspected IPA between June 1, 2014, and November 30, 2019, at the  Amsterdam University Medical Center, location VUmc. Patients who opted out of biobank storage were excluded.

### Microbiological and Chemical Testing on BALF

Fungal cultures were performed on Sabouraud dextrose agar (BD, Franklin Lakes, NJ, USA; Stock-Keeping Unit 274,720) with 50 mg/L chloramphenicol (Sigma, St-Louis, MO, USA; C-0378) incubated at 30 °C and 37 °C. Galactomannan index was measured with Platelia™ *Aspergillus* antigen tests (Biorad, Hercules, CA, USA). *Aspergillus* PCR was performed using an in-house real-time assay targeting the 28S rDNA gene [20, 21] and positive results were confirmed with the commercial CE-IVD assay AsperGenius® 1.0 (PathoNostics, Maastricht, The Netherlands). Samples were considered positive if both assays agreed. Iron levels were measured with the Cobas C502. Spectrophotometry with the FerroZine reagent was used on 100µL BALF after iron(III) was detached from transferrin using citric acid and reduced to iron(II) with ascorbic acid. The assay’s linearity was 0.3–85 µmol/L, with bilirubin, lipids, and hemolysis noted as interfering factors; no other inhibition controls were performed.

### Classification of IPA and Mortality

Clinical data on host factors and mycological tests were retrieved from electronic patient files. Chest CT abnormalities were initially described by the attending radiologist. If the classification according to EORTC/MSGERC 2020 criteria was not provided in the initial radiology report, it was added by two clinical investigators (HL and JH). Following EORTC/MSGERC 2020 definitions, patients were categorized as having proven, probable, possible, or no IPA. A sensitivity analysis was conducted using all available diagnostic results from the suspected IPA episode (such as follow-up BAL results) to account for false negatives. Data on all-cause mortality at 6 and 12 weeks post-BAL were collected, with causes of death categorized as IPA-related (either when IPA was the cause of death or contributed to the death of the patient), unrelated, or unknown. Since we previously found that the EORTC/MSGERC 2008 criteria correlated more strongly with 12-week mortality than the 2020 criteria [22], we also classified patients according to the 2008 criteria to explore potential correlations with BALF iron concentration.

### Statistics

Frequencies, proportions, and central tendencies were calculated and compared using either the Kruskal–Wallis Test or Wilcoxon signed-rank Test for non-parametric data. The Jonckheere-Terpstra test was used to assess trends between ordinal and continuous variables. Spearman’s rho (ρ) was used to evaluate quantitative correlations, with 0.21–0.40 indicating weak agreement, 0.41–0.60 moderate, 0.61–0.80 strong, and 0.81–1.00 very strong [23]. Receiver operating characteristic (ROC) curves were generated to calculate the area under the curve (AUC), with AUC values interpreted as: < 0.50 no discrimination, 0.50–0.69 poor, 0.70–0.79 acceptable, 0.80–0.89 excellent, and ≥ 0.90 outstanding discrimination [24]. Youden’s J-statistic (YI) was calculated from ROC curve coordinates. Multivariate logistic and Cox regression evaluated the effect of iron concentration and possible confounders on mortality over time. Kaplan–Meier curves displayed 12-week survival stratified by EORTC/MSGERC 2020 classification and iron levels, with pairwise comparisons using the Log Rank Test. Significance was set at a two-sided *p*-value of 0.05. Analyses were performed using IBM SPSS Statistics 28.0.1.1 and GraphPad Prism 9.2.1.

## Results

From a cohort of 444 episodes with suspected IPA from 273 patients, 126 samples could be retrieved for iron testing from the Amsterdam University Medical Centers biobank. We included 100 samples from 87 patients after excluding 7 samples with results below the assay’s detection limit and 19 samples deemed uninterpretable, even after retesting. The median patient age was 62.5 years (range 18–82, IQR 52–67), and 62% were male; for all baseline characteristics see Table 1. According to the EORTC/MSGERC 2020 criteria, 27% of patients were classified as probable IPA, 56% as possible IPA, and 17% as no IPA. Direct microscopy was negative in all 67 samples tested, while fungal culture was positive for *Aspergillus* in 1% (1/100) of samples, and the BALF galactomannan index was positive in 18.4% (18/98) of samples using the EORTC/MSGERC 2020 cut-off of 1.0. One patient was classified as probable IPA based on a positive serum galactomannan (6.30). *Aspergillus* PCR was positive in 27% of samples (27/100, cycle threshold cut-off ≤ 40.00), with 15 showing duplicate positive results. The median BALF iron concentration was 0.90 µmol/L (range 0.30–13.90, IQR 0.53–1.80). Details on the 13 samples from 11 patients that were included from either BAL at multiple locations during the same procedure or serial BAL at different time intervals are displayed in Supplementary Table 1, there was no significant difference between the repeated measurements (*p* = 0.593). The overall 6-week and 12-week all-cause mortality was 31% and 36%, respectively, with multiorgan failure due to (neutropenic) sepsis including respiratory failure due to bacterial, viral or fungal infection as the most common cause of death (22/36, 61.1%), compared to progression of the underlying hematologic malignancy (5/36, 13.9%) and other causes (9/36, 25.0%). For 6-week mortality 11/31 (35.5%) was IPA-related and for 12-week mortality 12/36 (33.3%).

**Table 1:** Baseline characteristics

	All samples (n = 100) Median (IQR) or *n* (%)
Age, y	62.5 (52.0–67.0)
Sex, male	62 (62.0)
*Underlying hematological malignancy*	
AML	50 (50.0)
MDS	22 (22.0)
ALL	1 (1.0)
Other	27 (27.0)
*HSCT*	
Allogeneic	37 (37.0)
Autologous	10 (10.0)
None	53 (53.0)
Neutropenia^1^	60 (60.0)
*Immunosuppressants*	
None	58 (58.0)
Corticosteroids	14 (14.0)
T-cell immunosuppressants	13 (13.0)
B-cell immunosuppressants	3 (3.0)
Corticosteroids and T-cell and/or B-cell immunosuppressants	12 (12.0)
Antifungal prophylaxis^2^	35 (35.0)
None	13 (13.0)
Fluconazole	18 (18.0)
Posaconazole	16 (16.0)
Itraconazole	1 (1.0)
Mould-active antifungal therapy^2^	52 (52.0)
Voriconazole	25 (25.0)
Posaconazole	2 (2.0)
Echinocandins	4 (4.0)
L-AmB	10 (10.0)
Combination therapy	9 (9.0)
Other	2 (2.0)
*Fungal diagnostics on BAL*	
Microscopy postivity^3^	0/67 (0)
Fungal culture positivity for *Aspergillus* species	1 (1.0)
GM positivity (≥ 1.0) GM ODI	18/98 (18.4) 0.20 (0.10–0.50)
*Aspergillus* PCR positivity (in duplicate) Ct-value positive PCR^4^	15 (15.0) 36.86 (35.87–37.77)
*EORTC/MSGERC 2020 classification*	
Proven IPA	0 (0)
Probable IPA	27 (27)
Possible IPA	56 (56)
No IPA/unclassifiable	17 (17)

If information was missing, we also reported the denominator for which the data was known within the cohort

ALL, acute lymphoblastic leukemia; AML, acute myeloid leukemia; BAL, bronchoalveolar lavage; EORTC/MSGERC, European Organization for Research and Treatment of Cancer and the Mycoses Study Group Education and Research Consortium; GM, galactomannan; HSCT, hematopoietic stem cell transplantation; IPA, invasive pulmonary aspergillosis; L-AmB, liposomal amphotericin B; MDS, myelodysplastic syndrome; ODI, optical density index; PCR, polymerase chain reaction

^1^ ≤ 0.5 × 10^9^/L for ≥ 10 days temporally related to episode

^2^In the last 24 hours before bronchoalveolar lavage

^3^Microscopic detection of fungal elements in BAL fluid indicating *Aspergillus* species

^4^Ct-values for positive in-house *Aspergillus* PCR

### Iron Concentration and IPA Classification According to EORTC/MSGERC 2020 Criteria

**Fig. 1:**
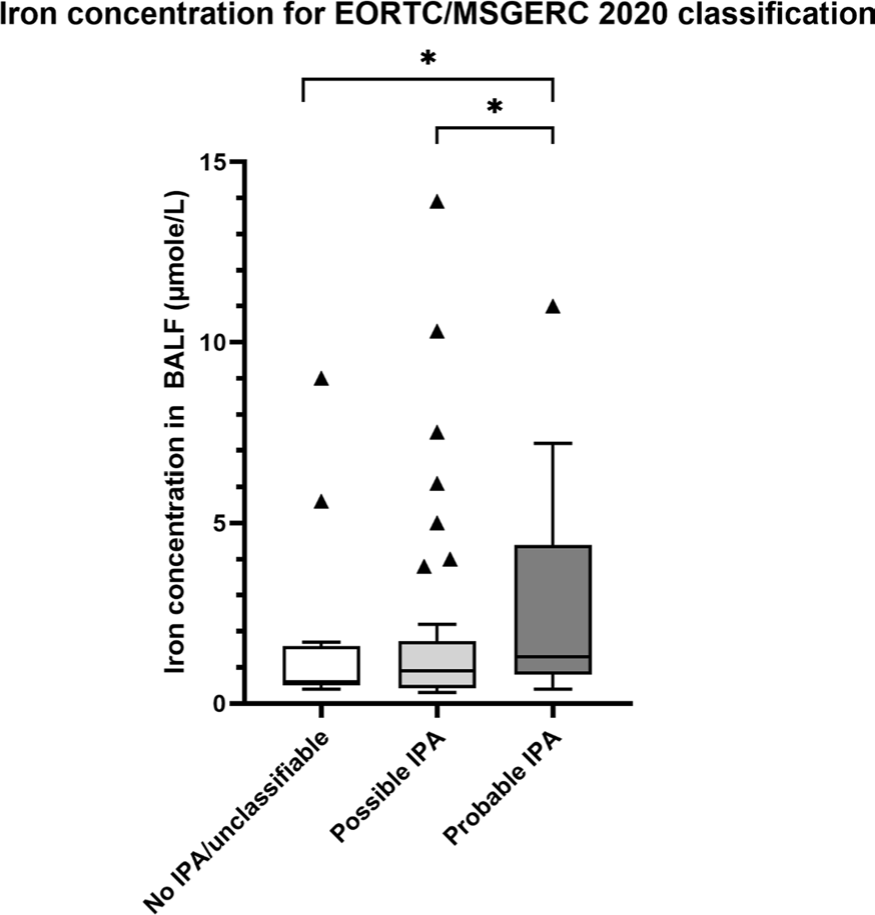
Differences in iron concentration in patients without IPA (Mdn 0.60, IQR 0.50–1.60, n = 17), with possible IPA (Mdn 0.90, IQR 0.43–1.73, n = 56) and with probable IPA (Mdn 1.30, IQR 0.80–4.40, n = 17) according to the EORTC/MSGERC 2020 consensus definitions. There is an increasing trend with *p* = 0.038

**Fig. 2:**
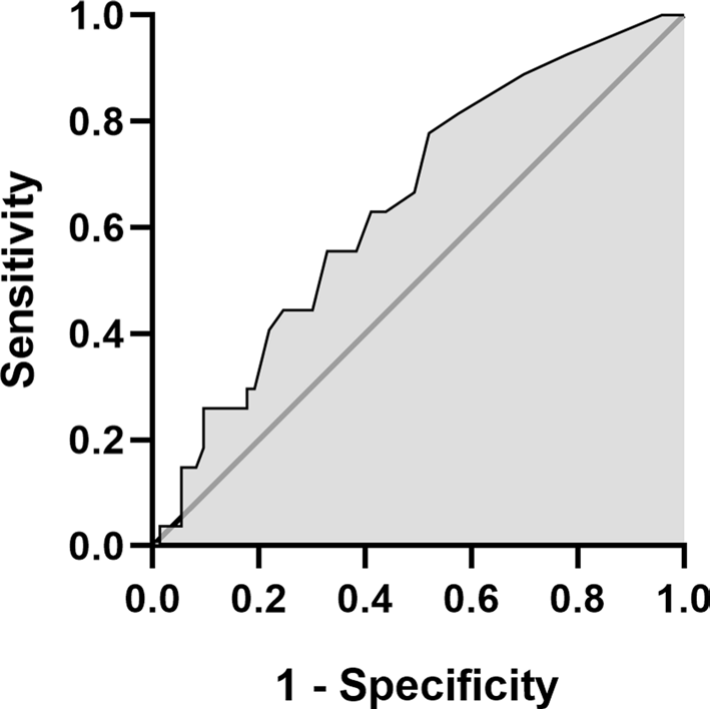
ROC curve for iron concentration and the discrimination of cases (proven or probable IPA, n = 27) and controls (possible or no IPA, n = 73) according to the EORTC/MSGERC 2020 definitions. The AUC was 0.648 (95% CI 0.531–0.764), *p* = 0.024

We assessed the potential of iron concentration as a diagnostic biomarker for IPA by correlating it with EORTC/MSGERC 2020 definitions. The median iron concentration was 1.30 µmol/L (IQR 0.80–4.40, n = 27) for probable IPA, 0.90 µmol/L (IQR 0.43–1.73, n = 56) for possible IPA, and 0.60 µmol/L (IQR 0.50–1.60, n = 17) for no IPA. A significant trend was observed with increasing iron levels from no IPA to probable IPA (Jonckheere-Terpstra Test, *p* = 0.038). Post-hoc comparisons showed significant differences between no IPA and probable IPA (*p* = 0.022), and between possible IPA and probable IPA (*p* = 0.021), see Fig. 1. ROC curve analysis indicated an area under the curve (AUC) of 0.648 (95% CI 0.531–0.764, *p* = 0.024), with an optimal cut-off iron concentration of 0.75 µmol/L to distinguish cases (proven and probable IPA) from controls (possible IPA and no IPA), yielding a sensitivity of 76.9% and specificity of 47.3%, see Fig. 2.

When evaluating iron concentration against EORTC/MSGERC 2008 classification, the median iron levels were 1.75 µmol/L (IQR 0.80–4.70, n = 22) for probable IPA, 0.90 µmol/L (IQR 0.50–1.43, n = 54) for possible IPA, and 0.60 µmol/L (0.50–1.70, n = 24) for no IPA, with a significant increasing trend between the groups (*p* = 0.014) and differences between no IPA and probable IPA (*p* = 0.007) and possible and probable IPA (*p* = 0.006). The ROC curve showed an AUC of 0.692 (95% CI 0.571–0.813, *p* = 0.006) and an optimal cut-off value of 1.25 µmol/L (sensitivity 63.6%, specificity 67.9%).

### Correlation Between BAL Iron Concentration and Fungal Load

**Fig. 3:**
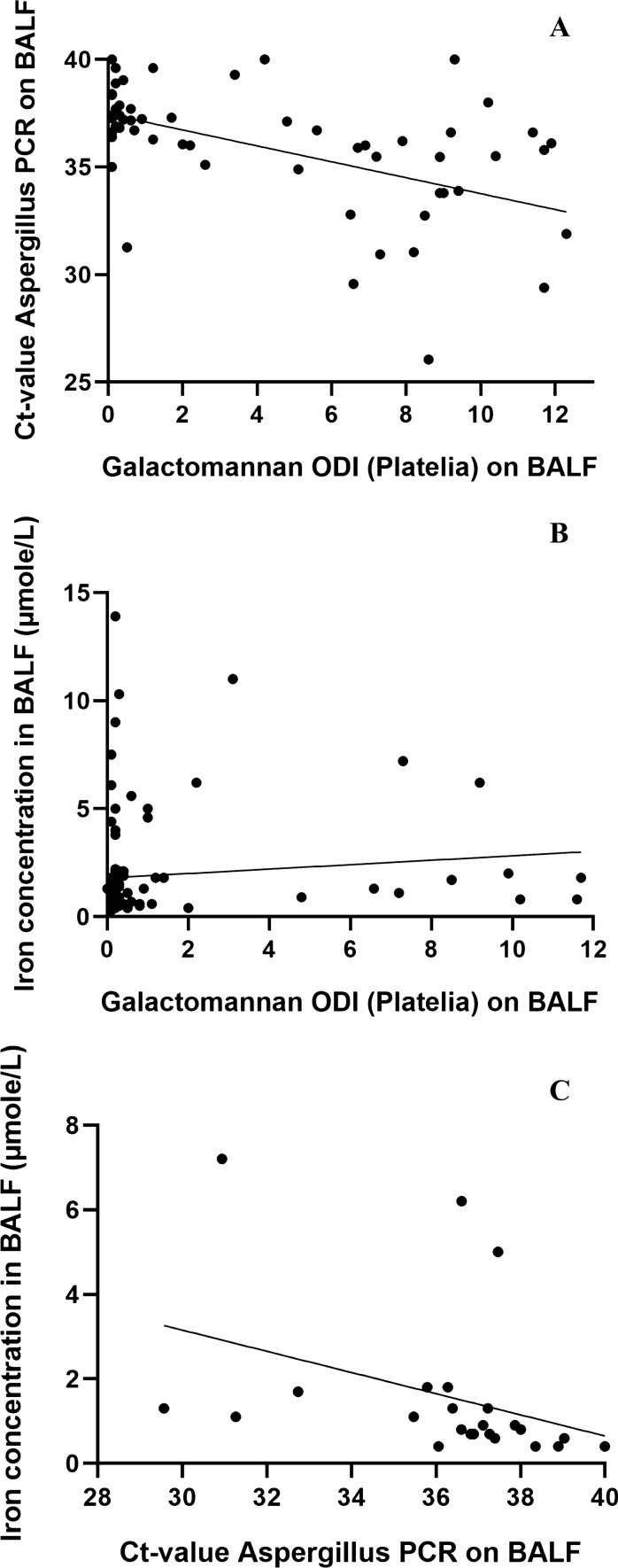
**A** Scatterplot for galactomannan ODIs and Ct-values of positive *Aspergillus* PCR on BAL fluid (Spearman’s ρ − 0.550 (95% CI − 0.710 to − 0.335), *p* < 0.001, n = 59). **B** Scatterplot for galactomannan ODIs and iron concentration on BAL fluid (Spearman’s ρ 0.323 (95% CI 0.127–0.494), *p* = 0.001, n = 98). **C** Scatterplot for Ct-values of positive *Aspergillus* PCR and iron concentration on BAL fluid (Spearman’s ρ − 0.602, 95% CI − 0.813 to − 0.250, *p* = 0.002, n = 24)

We examined the relationship between BALF iron concentration and fungal load, using galactomannan indices and *Aspergillus* PCR Ct-values. For the 59/444 samples with both galactomannan performed and PCR positive with Ct-value ≤ 40.00, we found an inverse correlation between galactomannan and PCR Ct-value (Spearman’s ρ − 0.550, 95%CI − 0.710 to − 0.335, *p* < 0.001) confirming both as indicators of fungal burden, see Fig. 3A. This was also the case in the subgroup of 24 BALF samples on which iron concentration too was tested with Spearman’s ρ − 0.511 (95% CI − 0.764 to − 0.124, *p* = 0.011).

BALF iron concentration correlated significantly with the galactomannan index (ρ 0.323, 95%CI 0.127–0.494, *p* = 0.001) in 98/100 samples, see Fig. 3B. Subgroup analysis revealed that this correlation was significant in patients with possible IPA (ρ 0.332, 95%CI 0.065 to0.555, *p* = 0.013, n = 55), but not in those with probable IPA (ρ 0.266, 95%CI −0.147 to 0.600, *p* = 0.189, n = 26) and in those without IPA (ρ −0.120, 95%CI −0.578 to 0.396, *p* = 0.646, n = 17).

BALF iron concentration also correlated strongly with Ct-values of positive *Aspergillus* PCR in 24 samples (ρ −0.602, 95%CI −0.813 to −0.250, *p* = 0.002), see Fig. 3C. This correlation was significant in probable IPA (ρ −0.534, 95% CI −0.813 to −0.057 *p* = 0.027, n = 17) but not in possible IPA (ρ −0.706, 95%CI −0.967 to 0.278, *p* = 0.117, n = 6). There was only one PCR-positive patient with no IPA (atypical abnormalities on HRCT, no mycological evidence except a single positive PCR with Ct-value 40.00).

In 29 patients (6 with probable IPA, 18 with possible IPA, and 5 without IPA), PCR on BALF detected at least one virus -8 cases of rhinovirus, 8 of parainfluenza virus, 4 of respiratory syncytial virus, 3 of influenza, 2 of herpes simplex type 1, and 1 case each of human metapneumovirus, cytomegalovirus, Epstein–Barr virus, and BK virus. The median Ct-value was 26.50 (IQR 23.30–30.85). There was no significant correlation between the viral PCR Ct-values and iron concentration (Spearman’s ρ −0.196, 95% CI −0.533 to 0.194, *p* = 0.307).

### Correlation Between BALF Iron Concentration and Mortality

Median iron concentration did not significantly differ between those who died within 6 weeks (Mdn 1.20 µmol/L, IQR 0.60–1.80, n = 31) and those who survived (Mdn 0.80 µmol/L, IQR 0.50–1.80, n = 69) with *p* = 0.229, or between those who died within 12 weeks (Mdn 1.20 µmol/L, IQR 0.63–1.98, n = 36) versus those who survived (Mdn 0.80 µmol/L, IQR 0.50–1.70, n = 64) with *p* = 0.114. Higher iron concentrations than the median of 0.90 µmol/L were associated with non-significant increases in 6-week (*p* = 0.157) and 12-week mortality (*p* = 0.137): 23.9% versus 37.0% and 28.3% versus 42.6%, respectively. Similarly, there were no significant differences observed for IPA-related deaths.

**Fig. 4:**
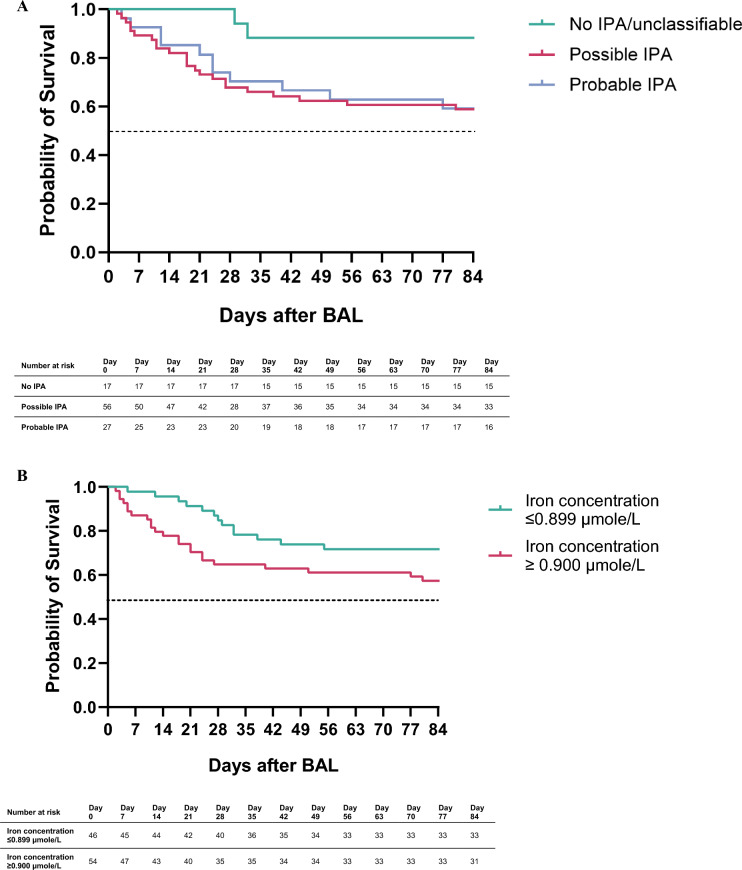
**A** Kaplan Meier curve for 12-week survival stratified to the EORTC/MSGERC 2020 classification. There was no difference in survival between possible and probable IPA (Log Rank (Mantel-Cox) = 0.026, *p* = 0.871). **B** Kaplan Meier curve for 12-week survival stratified to the median iron concentration in BAL fluid. There was a trend towards a difference in survival between patients with iron concentrations higher and lower than the median (Log Rank = 2.956, *p* = 0.086)

Kaplan–Meier analysis showed a significant difference in 12-week mortality between no IPA and probable IPA (*p* = 0.04), and between no IPA and possible IPA (*p* = 0.029), but not between possible and probable IPA (*p* = 0.871), see Fig. 4A. In contrast, higher iron concentrations did show a trend toward higher 12-week mortality (*p* = 0.086), see Fig. 4B. However, neither Cox proportional hazard regression nor multivariate logistic regression identified iron concentration as a significant predictor of mortality when corrected for presence of neutropenia, receipt of an autologous or allogeneic stem cell transplantation of the use of immunosuppressants, but the models were not statistically significant and showed a low goodness of fit.

### Correlation Between BAL Iron Concentration and Mortality in Patients with Probable IPA

In patients with probable IPA, there was a non-significant difference in iron concentration for 18 patients who were alive (Mdn 1.20 µmol/L, IQR 0.80–2.60) versus 9 patients who deceased within 6 weeks after BAL (Mdn 1.70 µmol/L, IQR 0.65–5.40), *p* = 0.705. After 12 weeks, there was a non-significant difference in iron concentration for 16 patients who were alive (Mdn 1.10 µmol/L, IQR 0.80–1.80) versus 11 patients who deceased (Mdn 1.80 µmol/L, IQR 0.70–6.20), *p* = 0.195. A Kaplan–Meier curve showed no significant survival difference between higher and lower iron concentrations than the median 0.90 µmole/L (Log Rank = 0.279, *p* = 0.598); the sensitivity analysis in which patients were classified according to all available diagnostic information did not change this (Log Rank = 0.090, *p* = 0.764).

## Discussion

In this study in patients with hematological malignancies and suspected IPA, we found a significant correlation between higher BALF iron concentrations and the likelihood of an IPA diagnosis based on EORTC/MSGERC 2020 criteria. However, iron concentration alone had insufficient discriminative power as a diagnostic marker, with a sensitivity of 76.9% and specificity of 47.3% at the optimal cut-off of 0.75 µmol/L, although it is comparable to the current microbiological tests for IPA on BALF [25, 26].

We also observed a correlation between BALF iron concentration and *Aspergillus* loads, as indicated by the GM index and Ct-values of *Aspergillus* PCR. Our previous findings revealed that the 2020 EORTC/MSGERC update led to a 22.6% reclassification of patients, including an 11.1% increase in those classified as probable IPA. The addition of PCR to the criteria might identify patients as probable IPA even when their actual risk of IPA was lower, as reflected by reduced 12-week mortality. This suggests that high Ct-values may indicate *Aspergillus* colonization rather than invasive disease [25], as was described by Imbert et al. in 2019 [27] and Huygens et al. in 2023 [28]. Higher iron concentrations, which are inversely correlated with Ct-values (as lower Ct-values portray higher DNA loads), may help distinguish between colonization and the risk of invasive growth.

We noted a trend towards higher 6-week and 12-week mortality with higher iron concentrations, though these differences were not statistically significant. This might be due to the retrospective cohort design, where sample size was not pre-determined, potentially leading to an underpowered analysis. Nonetheless, iron levels appeared to predict 12-week mortality more accurately (Log Rank (Mantel-Cox) = 2.956, *p* = 0.086) than the EORTC/MSGERC 2020 classification (Log Rank (Mantel-Cox) = 0.026, *p* = 0.871). This finding aligns with our previous publication, which also showed no difference in 12-week mortality between patients classified as possible IPA and probable IPA by EORTC/MSGERC 2020 [22]. Given the limited accuracy of individual tests, diagnosing IPA remains challenging, making the combination of multiple tests essential. Including iron concentration could enhance diagnostic effectiveness and provide added value.

The potential association between iron concentration, invasive disease, and mortality requires further investigation. Our hypothesis was that iron is a potentiating factor for *Aspergillus* virulence [11, 12], as higher iron concentrations likely portray higher levels of pulmonary microvascular and/or tissue damage through micro-hemorrhage and leakage of plasma proteins which could enable invasive growth of *Aspergillus* [18]*.* However, micro-hemorrhage and plasma protein leakage may independently predict poor outcomes, irrespective of fungal burden. Although we could not establish a direct causal mechanism due to the retrospective nature of our study, our data could support the idea that higher tissue iron concentrations might facilitate *Aspergillus* invasive growth, similar to mechanisms observed in lung transplant patients [18] and in rats where siderophore biosynthesis (i.e. microbial iron chelators) was essential for virulence of *Aspergillus* spp. [17]. This may explain the association between high BALF iron concentrations and probable IPA, high BALF galactomannan levels, low PCR Ct-values, and possibly higher 12-week mortality. Alternatively, elevated BALF iron could result from major hemorrhage, severe pancytopenia, or possibly from factors like smoking or underlying structural lung conditions, as various studies have highlighted the role of iron in the pathogenesis of these diseases [29–31]. Prospective animal studies with serial BALF analyses should investigate the causal relationship between iron concentrations, IPA risk, and mortality, as well as the potential effect of intrapulmonary iron chelation therapy.

A limitation of this study was the lack of standardization in BAL procedures, as factors like the amount of recovery fluid may affect concentrations of IPA markers, including galactomannan, *Aspergillus* DNA and possibly iron. Another limitation is that BALF iron concentration indirectly measures tissue iron, and the correlation between these parameters is not well established. Future research should explore more direct markers, such as fungal siderophores [32], and consider factors like serum iron, hepcidin levels, and blood transfusions, which also influence tissue iron levels [33, 34]. While previous studies found a poor correlation between most serum iron markers and invasive fungal infections, a decrease in total iron-binding capacity was associated with more infections [35–37]. It would be interesting to measure this parameter in prospective studies to better understand its potential role in predicting and managing invasive fungal infections.

In conclusion, while BALF iron concentration appears to correlate with a higher likelihood of IPA according to the EORTC/MSGERC 2020 criteria, it should not be regarded as a definitive standalone diagnostic marker. Elevated iron concentrations are associated with higher *Aspergillus* fungal loads and may be linked to increased 12-week mortality. However, given the various potential confounding factors, further prospective studies are essential to establish causality. These findings warrant additional investigation into BALF iron as a potential marker for 12-week survival, but validation is necessary before considering it as a supplementary marker in the current EORTC/MSGERC 2020 classification for probable or possible IPA.

## Supplementary Information

Below is the link to the electronic supplementary material.

Supplementary file1 (DOCX 33 KB)
