# Publisher Correction: Iron Levels in Bronchoalveolar Lavage Fluid of Hematological Patients with Suspected Invasive Pulmonary Aspergillosis and their Association with 12‑week Mortality: A Retrospective Cohort Study

**DOI:** 10.1007/s11046-025-00938-6

**Published:** 2025-03-21

**Authors:** H. Lamberink, J. Heijmans, A. Wagemakers, K. van Dijk

**Affiliations:** 1 Department of Medical Microbiology and Infection Prevention, Amsterdam University Medical Centers, Amsterdam, The Netherlands; 2 Department of Hematology, Amsterdam University Medical Centers, Amsterdam, The Netherlands; 3 Department of Medical Microbiology and Infection Prevention, Onze Lieve Vrouwe Gasthuis, Amsterdam, The Netherlands

**Correction to: Mycopathologia (2025) 190:23** 10.1007/s11046-025-00934-w

In the discussion section of this article, in the sentence ‘This finding aligns with our previous publication, which also showed no difference in 12-week mortality between patients classified as possible IPA and probable IPA by EORTC/MSGERC 2020 [25]’, the 25th reference ‘Boch et al. (2016)’ was cited incorrectly instead of 22nd reference ‘Lamberink et al. (2022)’. The correct sentence should read as ‘This finding aligns with our previous publication, which also showed no difference in 12-week mortality between patients classified as possible IPA and probable IPA by EORTC/MSGERC 2020 [22]’.

The original article has been corrected.

