# Supplementary material for: Iron Levels in Bronchoalveolar Lavage Fluid of Hematological Patients with Suspected Invasive Pulmonary Aspergillosis and their Association with 12-week Mortality: A Retrospective Cohort Study
Source: Mycopathologia. 2025 Feb 3;190(1):23. doi: 10.1007/s11046-025-00934-w (PMC11790682; doi:10.1007/s11046-025-00934-w)
Supplement: Supplementary file 1 — Supplementary file1 (DOCX 33 KB) [file 11046_2025_934_MOESM1_ESM.docx]

|  | | | | | | **BAL fluid results** | | | |  | |
| --- | --- | --- | --- | --- | --- | --- | --- | --- | --- | --- | --- |
| **Patient no.** | **Timing of BAL** | **Location of BAL** | **Underlying disease** | **Dominant radiological finding** | **Iron concentration** | **Direct microscopy** | **Fungal culture** | **GM ODI** | ***Aspergillus* PCR (lowest Ct-value)** | **EORTC/MSGERC 2020 classification^1^** | **State at 12 weeks (days after BAL)** |
| 1 | Day 1 | LUL | MDS | WSC | 0.900 | NA | Negative | 0.30 | Negative | Possible IPA | Alive |
|  | Day 1 | LUL | MDS | WSC | 0.400 | NA | Negative | 0.20 | Negative | Possible IPA | Alive |
|  | Day 20 | RLL | MDS | WSC | 6.20 | NA | Negative | 2.20 | Negative | Probable IPA | Alive |
| 2 | Day 1 | RLL | CMML | Halo sign | 6.20 | NA | Negative | 9.20 | Single positive^2^ (36.61) | Probable IPA^3^ | Deceased (day 12) |
|  | Day 1 | RLL | CMML | Halo sign | 0.500 | NA | Negative | NA | Negative | Probable IPA^3^ | Deceased (day 12) |
| 3 | Day 1 | RML | MDS-MLD | WSC | 0.800 | NA | Negative | 0.10 | Positive in duplicate (36.60) | Probable IPA | Alive |
|  | Day 6 | RML | MDS-MLD | WSC | 7.50 | Negative | Negative | 0.10 | Negative | Possible IPA | Alive |
| 4 | Day 1 | Unknown | MDS-EB2 | Nodules | 0.400 | Negative | Negative | 0.10 | Single positive^2^ (38.36) | Possible IPA | Alive |
|  | Day 13 | Unknown | MDS-EB2 | Nodules | 0.800 | Negative | Negative | 11.60 | Negative | Probable IPA | Alive |
| 5 | Day 1 | LUL | MDS | Nodules | 1.50 | NA | Negative | 0.10 | Negative | Possible IPA | Deceased (day 18) |
|  | Day 14 | LUL | MDS | Nodules | 1.00 | NA | Negative | 0.20 | Negative | Possible IPA | Deceased (day 5) |
| 6 | Day 1 | LUL | MDS-EB2 | Nodules | 4.60 | Negative | Negative | 1.00 | Negative | Probable IPA | Deceased (day 40) |
|  | Day 21 | LUL | MDS-EB2 | Nodules | 0.600 | Negative | *C. glabrata* | 0.10 | Negative | Possible IPA | Deceased (day 20) |
| 7 | Day 1 | LUL | AML | Nodules | 1.20 | Negative | Negative | 0.10 | Negative | Possible IPA | Alive |
|  | Day 21 | RUL | AML | Nodules | 0.400 | Negative | Negative | 0.10 | Negative | Possible IPA | Alive |
| 8 | Day 1 | RUL | MCL | WSC | 1.10 | Negative | Negative | 0.20 | Negative | Possible IPA | Deceased (day 84) |
|  | Day 35 | LLL | MCL | WSC | 11.00 | Negative | Negative | 3.10 | Negative | Probable IPA | Deceased (day 51) |
| 9 | Day 1 | Unknown | MDS | Nodules | 0.40 | Negative | Negative | 0.10 | Negative | Possible IPA | Alive |
|  | Day 50 | RLL | MDS | Nodules | 0.70 | NA | Negative | 0.60 | Negative | Possible IPA | Alive |
| 10 | Day 1 | RML | AML | Halo sign | 1.30 | Negative | Negative | 0.90 | Positive in duplicate (37.23) | Probable IPA | Alive |
|  | Day 64 | RML | AML | Halo sign | 0.80 | Negative | Negative | 0.20 | Negative | Possible IPA | Deceased (day 32) |
|  | Day 75 | RML | AML | Nodules | 0.90 | Negative | Negative | 0.10 | Negative | Possible IPA | Deceased (day 21) |
| 11 | Day 1 | RLL | AML | Halo sign | 0.40 | Negative | Negative | 0.10 | Negative | Possible IPA | Alive |
|  | Day 454 | LUL | AITL | Nodules | 0.50 | Negative | Negative | 0.20 | Negative | Possible IPA | Alive |

**Supplementary Table 1. Details of 11 patients who underwent BAL at multiple locations on the same day or serial BAL at different intervals in time.**

1: The EORTC/MSGERC 2020 criteria are applied to the individual samples included, not for all diagnostics performed during the episode.
2: AsperGenius® not performed.

3: Probable IPA based on serum GM 6.30.

Abbreviations: AITL = angioimmunoblastic T-cell lymphoma; AML = acute myeloid leukemia; BAL = bronchoalveolar lavage; CMML: chronic myelomonocytic leukemia; EORTC/MSGERC = European Organization for Research and Treatment of Cancer and the Mycoses Study Group Education and Research Consortium; GM = galactomannan; IPA = invasive pulmonary aspergillosis; LLL = left lower lobe; LUL = left upper lobe; MCL = mantle cell lymphoma; MDS = myelodysplastic syndrome; MDS-EB2 = myelodysplastic syndrome with excess blasts; NA = not assessed; ODI = optical density index; PCR = polymerase chain reaction; RLL = right lower lobe; RML = right middle lobe; RUL = right upper lobe; WSC = wedge-shaped consolidation.
